# Comparative and Developmental Anatomy of Cardiac Lymphatics

**DOI:** 10.1155/2014/183170

**Published:** 2014-01-27

**Authors:** A. Ratajska, G. Gula, A. Flaht-Zabost, E. Czarnowska, B. Ciszek, E. Jankowska-Steifer, J. Niderla-Bielinska, D. Radomska-Lesniewska

**Affiliations:** ^1^Department of Pathology, Medical University of Warsaw, Chałubińskiego 5, 02-004 Warsaw, Poland; ^2^Student Scientific Group at the Department of Pathology, Medical University of Warsaw, Chałubińskiego 5, 02-004 Warsaw, Poland; ^3^Department of Pathology, Children's Memorial Health Institute, Aleja Dzieci Polskich 20, 04-730 Warsaw, Poland; ^4^Department of Clinical Anatomy, Medical University of Warsaw, Chałubińskiego 5, 02-004 Warsaw, Poland; ^5^Department of Histology and Embryology, Medical University of Warsaw, Chałubińskiego 5, 02-004 Warsaw, Poland

## Abstract

The role of the cardiac lymphatic system has been recently appreciated since lymphatic disturbances take part in various heart pathologies. This review presents the current knowledge about normal anatomy and structure of lymphatics and their prenatal development for a better understanding of the proper functioning of this system in relation to coronary circulation. Lymphatics of the heart consist of terminal capillaries of various diameters, capillary plexuses that drain continuously subendocardial, myocardial, and subepicardial areas, and draining (collecting) vessels that lead the lymph out of the heart. There are interspecies differences in the distribution of lymphatic capillaries, especially near the valves, as well as differences in the routes and number of draining vessels. In some species, subendocardial areas contain fewer lymphatic capillaries as compared to subepicardial parts of the heart. In all species there is at least one collector vessel draining lymph from the subepicardial plexuses and running along the anterior interventricular septum under the left auricle and further along the pulmonary trunk outside the heart and terminating in the right venous angle. The second collector assumes a different route in various species. In most mammalian species the collectors run along major branches of coronary arteries, have valves and a discontinuous layer of smooth muscle cells.

## 1. Introduction

The existence and importance of cardiac lymphatics has been neglected for many years. Recently more attention has been devoted to this system since it was discovered that besides helping maintain intramyocardial pressure and preventing tissue edema it plays an important role in many heart pathologies, such as atherosclerosis and interstitial fibrosis [1]. Lymphatics residing in the adventitial layer of atherosclerotic vessels can drain fluid and inflammatory molecules and cells to local nodes. This is how blood vessels interact with the lymphatic system [2]. Impairment of lymph flow has been reported to be associated with coronary artery injury in human and animal hearts [3, 4] as well as with other pathologies, such as enhanced tissue damage followed by necrosis and increased interstitial fibrosis accompanied by fibroblast activation and proliferation in human and canine hearts as well as accumulation of myxoid material consisting of hyaluronic acid and chondroitin sulphates in the canine tricuspid valve [5–7]. Surgery-induced damage to adventitial fat pad lymphatics located around the aortic roots near the sinoatrial node has been reported to lead to atrial fibrillation and heart dysfunction [8]. Apart from conduction disturbances, poor myocardial contraction has also been reported [4, 7]. In fact, an obstruction of lymph flow through any body organ predisposes to inflammation and/or fibrous tissue formation as well as tissue edema.

Nowadays, due to improved techniques that make it possible to distinguish blood vessels from lymphatics, the lymphatic system is no longer considered as secondary to the blood circulatory system [9, 10]. Furthermore, human myocardial lymphatics have been described in detail with regard to their location and structure, and a marked variability of the lymphatic outflow paths (or lymphatic collectors) in human and animal hearts has been reported [11, 12]. However, formation of lymphatic vessels during heart development has not been entirely elucidated, partially due to a lack of a specific marker for visualization of endothelial lymphatics.

Knowledge of the structure and location of capillary lymphatic system and major draining branches in the heart in various species as well as its relationship with the circulatory system would help in understanding the proper functioning of the heart. Additionally, due to an immense development of modern technologies and the use of transgenic animals in many research tasks, a better understanding of normal cardiac lymphatic vessel embryogenesis is also in progress. Thus, the aim of this paper is to summarize the current knowledge on cardiac lymphatic anatomy in various species and discuss the basic steps of lymphatic vessel development and maturation.

## 2. Gross and Microscopic Visualization of Cardiac Lymphatics

The techniques for visualization of gross anatomy of lymphatics vary. A useful method to demonstrate location of lymphatics is to inject India-ink to subepicardium, myocardium, or subendocardium, areas of the conduction system and atrioventricular valves, via a needle, glass pipette, or syringe. Additionally, other filling substances have been used by investigators in the past such as radiopaque iodized oil, latex or vital dye (trypan blue), Evans blue, resins, micropulverized barium sulfate, and carbon (very small carbon particles) combined with polyvinylpyrrolidone [1, 13–18]. Another technique that has been used in some studies was to immerse fresh or 4% paraformaldehyde-fixed hearts in 0.5–1% hydrogen peroxide for a few minutes. Hydrogen peroxide initiates oxidoreduction with catalase or peroxidase in tissue or lymph, producing oxygen and water. The released oxygen causes distention of lymphatics and sometimes blood vessels [19]. This particular method, that is, hydrogen peroxide injection, has been recommended for better visualization of endocardial lymphatics [11, 20]. Additionally, it is advisable to inject silver nitrate to coronary blood system. In such hearts, lymphatic endothelial cells are easily identified based on their “leaf-oak” shape. An unusual model to study the lymphatics in the heart has been proposed and it involves induction of *myocarditis* with the use of Trypanosoma. Due to its strong affinity to lymphatic vessels, this parasite made it easier to study the morphology and confirm the appearance of lymphatic capillaries, for example, in valves or papillary muscles [21].

Nowadays, modern techniques allow lymphatic endothelial cells (LEC) with specific markers to be easily demonstrated in various species on histological, confocal microscope sections and by the use of the “whole mount” immunohistochemical method [22–25]. For detection of LEC it is recommended to demonstrate at least two markers simultaneously, such as Lyve-1 and Prox-1. In addition, it has been reported that lymphatic endothelial cells express transcription factor Sox18, VEGFR3, neuropilin-2, Tie-2, podoplanin, VE-cadherin (=CD144), Flk-1, and other markers, however, that are not exclusively specific for LEC. Many studies emphasize that LEC are negative for CD34 and vWF; however, in some tissues, for example, in an inflamed dental pulp, LEC possess vWF particles that are concentrated in Weibel-Palade bodies [26, 27]. Also the bovine thoracic trunk endothelium has been reported to contain vWF particles [26]. It has not been demonstrated whether the presence of vWF is always related with inflammatory LEC in other tissues and organs.

## 3. Interspecies Variations in Cardiac Lymphatics

### 3.1. Capillary Plexuses

The earliest detailed data on cardiac lymphatics has been described by Patek [13] who observed the lymphatics of a dog heart as a 3D lymphatic plexus located in the connective tissue between the epicardium and endomyocardium. He used the India-ink injection method by introducing the India-ink directly into the beating myocardial wall of living animals to study lymphatics in thick, cleared sections. This lymphatic location has been confirmed by Johnson and Blake [28] who used hydrogen peroxide to grossly visualize cardiac lymphatics of humans, pigs, and dogs. Further studies in most species, including humans, revealed that lymphatic capillaries were located uniformly in the myocardial, subepicardial, and subendocardial areas forming a continuous plexus [29, 30]. It has been reported, however, that in some species, for example, in rabbit [31] and in mouse hearts [32] lymphatics of the subepicardial areas and of the outer layer of the myocardial wall were more dense as compared to lymphatics of the subendocardial area. Some authors have reported that the subendocardial lymphatics were even absent in those species. The capillaries were of 15–20 *μ*m in diameter and joined larger channels of 60 to 100 *μ*m. Those channels coursed in the superior part of the interventricular septum parallel to the atrioventricular sulcus. The apices of papillary muscles were penetrated by lymphatic plexuses of 30–45 *μ*m with occasional channel vessels of 150 *μ*m. In dog hearts, these lymphatic vessels formed a denser network than in the pig heart. The human subepicardial plexus showed a similar pattern to that of the canine and porcine capillary plexuses [28] and coursed over the atrial and the ventricular surfaces with certain minor differences. Lymphatics of canine and porcine hearts were present on the atrial surfaces along the tricuspid and mitral valves, directed towards the annuli where they merged with a larger channel of 110–225 *μ*m in diameter; however, their further courses were not determined [28]. The ventricular surfaces of atrioventricular valves were devoid of lymphatic vessels [19, 28]. Atrioventricular valves contain no blood capillaries except in their proximal portions. In contrast, there are lymphatics draining the valves; the richest lymphatic plexus can be found in the anterior cusp of the mitral valve. The network of lymphatic capillaries in the mitral valve of a dog heart tends to increase when lymphatic flow is obstructed [33]. The lymphatics of the human cardiac mitral valve play an important role in the consequences of rheumatic fever, since this disease has an impact on adjacent tissues lined with endothelium such as lymphatics, blood vessels, and the endocardium [33]. In human hearts the presence of lymphatics has been demonstrated only in the mitral valve, whereas in the tricuspid valve lymphatics were absent. It is not known whether the lack of lymphatics in the tricuspid valve may have been caused by technical problems, whether it depends on species specificity, and/or whether it is typical only for human hearts. Lymphatics were not found in the semilunar valves of the aorta and the pulmonary trunk of human, pig and dog hearts [17, 28, 33, 34].

Shimada and colleagues noted that the lymphatic plexus was less extensive in the atria than in the ventricles [19]. In the atrial wall lymphatics of the subepicardial space were scant, whereas in the subendocardial area, they were completely absent in various species, including rabbits [19, 31].

The capillary lymphatic system has been demonstrated by various techniques to be present in the conduction tissue of mammalian hearts: in the sinoatrial node of man, dog, and rabbit [15, 16, 27, 34, 35], in the atrioventricular node of man, dog, and rabbit [16, 34–36], in the His bundle of man and rabbit, in the right bundle branch of dog [37], in the left bundle branch of sheep, and in the Purkinje fibers of the moderator band in sheep [38]. The lymphatic plexus of the sinoatrial node of the human heart drain lymph via the subepicardial lymphatic network of the right atrium directly to the right main trunk or indirectly (via lymphatic vessel of the posterior edge of the right auricle) to the right main trunk. The lymphatic plexus of the atrioventricular node and the atrioventricular bundle drive lymph to the left main trunk [16]. The lymphatic network carrying lymph from the conduction system belongs to lymphatic capillaries of the myocardial plexus and directs lymph to the subendocardial and/or to the subepicardial plexuses and subsequently to the subepicardial collectors [16]. The vessels of the subendocardial ventricular lymphatic plexus drain to the left main trunk.

Adventitial lymphatics have been reported to be rather extensive along the aorta as well as along the coronary arteries in mammalian species. Our work indicated that by the end of the prenatal life an extensive adventitial lymphatic plexus was developed at the roots of the aorta and the pulmonary trunk in mouse hearts [23]. In the human heart the lymphatic plexus has been also described to be located along the coronary arteries and in the adventitia of the aorta [38]. Impairment of lymph drainage in the adventitia of large arteries plays an important role in the pathogenesis of atherosclerosis and arterial wall disease [39]. An intimal edema is associated with atherosclerotic plaque formation [40, 41].

The myocardial lymphatic capillaries were less numerous as compared to blood capillaries. The ventricular myocardium of cat, rabbit, and human hearts possessed an average of 1,029 blood capillaries per 1,000 muscle cells ([42] cited after [19]), whereas in a dog heart there was only 1 lymphatic capillary per 20–30 muscle cells ([43] cited after [19]).

In some species, only fragments of myocardium were studied. For example, some authors studied the myocardium of the septomarginal band in lamb hearts [38]. Lymphatic capillaries in the septomarginal band did not present earlier than in 143-day-old fetuses, and then, the plexus developed further in postnatal and adult lambs.

### 3.2. Terminal Vessels (Capillaries)

Terminal vessels (lymphatic capillaries) were irregular in shape and diameter, possessed many branches, and exhibited tortuous courses. The caliber of lymphatic capillaries varied, depending on the author, animal species, or technique used, and ranged from 20 to 100 *μ*m [19] or even from 30 to 300 *μ*m [30] or larger—up to 400 *μ*m. Lymphatic capillaries and drainage vessels often formed the networks and meshes that were irregular in contour. Lymphatic capillaries can be found within interfascicular connective tissue surrounding muscle bundles and accompanying blood vessels. Lymphatic capillaries of the subendocardium are localized within a single plane parallel to the endocardial surface. They were located in the loose connective tissue that was situated between the lining endothelium and Purkinje fibers. In the human heart, the subendocardial lymphatic capillaries are less pronounced. Their diameters measure between 20 and 45 *μ*m with occasional channels of up to 150 *μ*m. The appearance of human endocardial lymphatics is similar to that of canine and porcine heart.

### 3.3. Collectors

The smaller lymphatic capillaries of the myocardial plexus converged forming larger drainage lymphatic vessels that contained valves. These drainage vessels coursed in the subepicardial space [19]. In the human heart, drainage vessels (collectors) composed of the left and right lymphatic trunks course along the periadventitial area of the major coronary artery branches [19, 44]. A similar pattern has been described in canine and porcine hearts. The left trunk (so called the major trunk) was shown to collect lymph from the anterior interventricular and the left marginal branch as well as from the posterior interventricular branch merging into the left coronary trunk. This trunk passed behind the left atrial appendage subsequently ascended onto the posterior surface of the pulmonary trunk and up to the pretracheal nodes at the level of the aortic arch. From this node a single vessel passed cephalad to the right behind the aorta, to the cardiac lymph node which lay between the superior vena cava and the right brachiocephalic artery. Usually, this lymphatic channel branches into two or more vessels before it reaches the cardiac lymph node, from where a variable number of channels passed upwards (cephalad) to the right lymphatic duct that terminate in the right venous angle [44, 45]. The right trunk (the right coronary trunk, so called the minor, or lesser trunk) was found to collect lymph from the right territory of the heart, that is, from the right ventricle, proceed on the anterior surface of the aorta, enter the left anterior mediastinal chain and left paratracheal lymph nodes, pass towards the upper reaches of the thoracic trunk [45], and terminate in the left venous angle [44]. According to Feola et al. [46], who injected human cadaveric hearts with Evans Blue (T1824 dye), both the left and the right trunks unified as one main lymphatic vessel toward the root of the aorta; this pattern was observed in 7 out of 9 cadaveric human hearts. In 2 cases, these branches remained separate (the left and the right coronary lymphatic branches): one collector running along the left ascending branch of the coronary artery and the other one running along the main branch of the right coronary artery. The results by Feola et al. [46] might indicate that a variability of collector vessel courses exists in human hearts. Collecting vessels of the human heart were situated in the subepicardium; their walls were thin, consisted of endothelium supported by elastic fibers anchored to the adjacent connective tissue, and contained a few smooth muscle cells [11]. Some authors discern precollectors and collecting vessels (major trunks) [47]. The difference between them was an irregular and discontinuous smooth muscle layer. The point along the course of precollectors where smooth muscle cells appeared in their walls has not been identified; however, it probably lay within the epicardium. The precollectors were often smaller in diameter than the absorbing lymphatics draining lymph from the subendocardium to the subepicardium. A three-dimensional reconstruction of two precollectors indicated that these two vessels having irregular walls joined and then separated again showing an irregular, discontinuous arrangement of musculature [47]. Stretches completely devoid of smooth muscle cells alternated with stretches with abundant musculature [47]. Precollectors and collecting vessels varied in size and form, but no relationship could be found between the vessel size and their form. Smaller-sized vessels had sometimes more musculature, whereas larger vessels possessed long stretches devoid of smooth muscle cells.

In Macaca monkeys the pattern of cardiac lymphatics has been reported to be comparable to that of man, dog, and pig [3]. Due to a technical approach one can distinguish one or two branches of collecting vessels. The principal ascending draining lymphatic vessel located in the interventricular septum and later positioned near the left atrial appendage was constantly visible regardless of the method applied; however, in old monkeys this vessel could be sclerotic and highly beaded in appearance.

**Figure 1:**
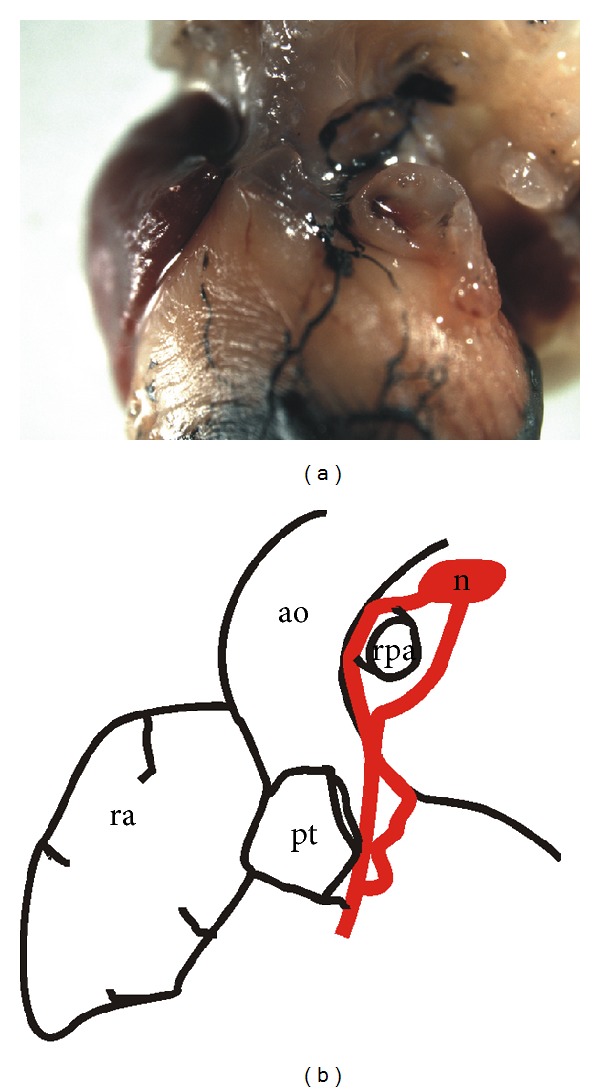
(a) A photography of an India-ink-injected adult mouse heart demonstrating the structure and the route of the major lymphatic collector; (b) a scheme showing the extracardiac route of this collector terminating in a regional lymph node (in red); ra—right auricle; pt—pulmonary trunk; rpa—right pulmonary artery; ao—aorta.

The structure and course of precollectors and collecting subepicardial vessels were demonstrated in our lab by injecting mouse hearts with India ink mixed with gelatin and showing their course as dividing branches and merging branches. These vessels vary in size and diameter along their course. The left major collector in murine hearts corresponds to the major cardiac trunk in other mammalian species. It drains the paraconal interventricular sulcus around the left conal vein towards the left side of the pulmonary trunk and upwards to the mediastinum via the nearest lymph node (corresponding to the tracheobronchial node in other species) (Figure 1). The second collector vessel of the mouse heart can be seen on the atrial surface with the use of various techniques. It can also be discerned in fetal hearts by the end of prenatal life on the dorsal aspect of the heart. This vessel drains the lymph from the left ventricle running along the left cardiac vein and subsequently cephalad on the coronary sinus, on the atrial surface, and then upwards towards the mediastinum.

Contrary to human, pig, and dog heart where major lymphatic precollectors/collectors accompany coronary artery branches, in the mouse and rat hearts these vessels accompany branches of cardiac veins (are in a close proximity to major cardiac veins).

## 4. Functional Anatomy of Cardiac Lymphatics

Patek [13] and others suggested that lymph flows from the subendocardial to the subepicardial lymphatic plexus and subsequently leaves the heart via lymphatic trunks draining to the regional lymph nodes. It has been demonstrated as a confirmation that colloidal particles injected into myocardium appeared quickly (only a few seconds) within the lymphatics of the subepicardial area.

Cardiac lymphatic flow has been suggested to be propelled by a passive pumping [4], for example, by myocardial contractions. During diastole, the pressure of the blood in the ventricles drives lymph from the subendocardial to the myocardial lymphatics, whereas during systole myocardial contraction forces lymph from the myocardial lymphatic vessels to the subepicardial lymphatics [4].

## 5. Developmental Anatomy of Heart Lymphatics

The development of cardiac lymphatic vessels has been the subject of a few studies. As development of lymphatics commonly followed blood vessel formation in the body of the embryo, the same sequence was observed in developing organs, such as the heart; that is, the lymphatics occurred later than blood vessels. The species studied were rats, mice, and birds such as quail and chicken [22–25].

**Figure 2:**
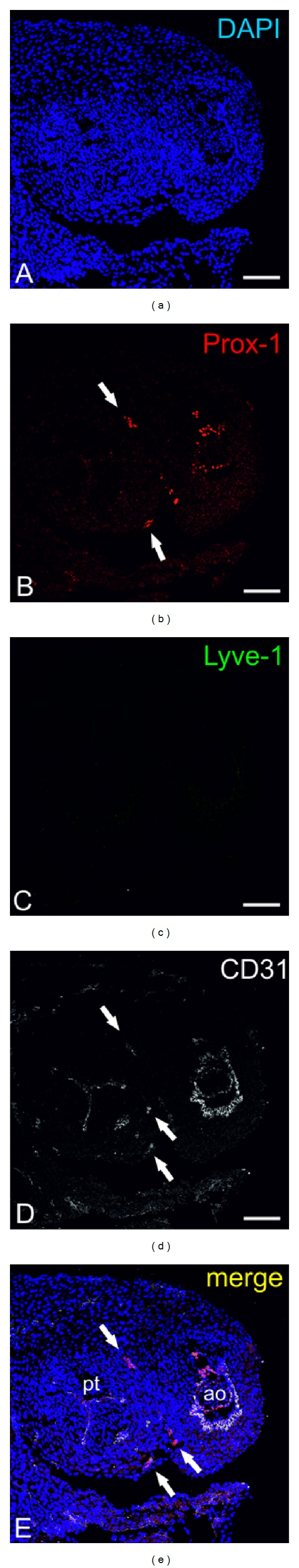
Confocal microscopy images of a 13-dpc mouse heart stained with DAPI (blue), anti-Prox-1 (red), anti-Lyve-1 (green), and anti-CD31 (white). Prox-1^+^/CD31^+^/Lyve-1^−^ vessels (white arrows) are present along the great arteries; ao—aorta; pt—pulmonary trunk; scale bars = 100 *μ*m.

**Figure 3:**
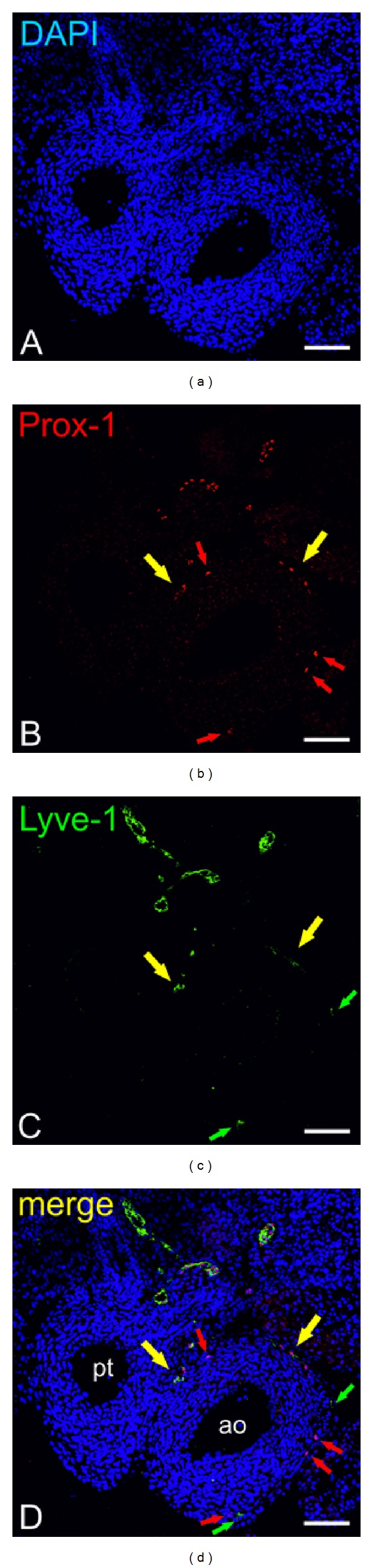
Confocal microscopy images of a 14-dpc mouse heart stained with DAPI (blue), anti-Prox-1 (red), and anti-Lyve-1 (green). Lyve-1-positive cells (green arrows), Prox-1-positive cells (red arrows), and Lyve-1/Prox-1-double positive vessels (yellow arrows) are visible along the great arteries; ao—aorta; pt—pulmonary trunk; scale bars = 100 µm.

Development and structure of lymphatics in the avian heart (chicken, quail) have been studied fragmentarily. Only a few papers dealing with injection techniques and with the use of lymphatic markers have been published [22, 24, 48, 49]. Using Prox-1, Lyve-1, and QH1 markers for lymphatic endothelium, the major trunks of lymphatics were found along the great arteries. These trunks collect lymph from the subepicardial plexus. During heart development lymphatic vessel formation proceeded from the base to the apex, similar to mouse and other mammal hearts [22]. The proepicardium has been excluded as a source of lymphangioblasts homing to the developing heart [22]; however, there is no consistency on whether cardiac lymphatic endothelial cells might stem by sprouting and/or budding from veins. In a homotropic grafting experiment in which quail PE cells were grafted to chicken hearts it has been found that some Prox-1-positive lymphatic trunks (along the great arteries) contain QH1-positive endothelial cells (derived from quail). This means that some lymphatics form lymphovenous anastomoses. This fact might suggest that lymphatics at the base of the great arteries might derive by ingrowth of venous endothelial cells to a lymphatic trunk. Similar lymphovenous anastomoses were described in pig and human adult hearts [50]. Our study in mouse fetuses demonstrates that first Lyve-1-bearing cells and tubules occurred on the dorsal atrioventricular sulcus and subsequently these structures gained the Prox-1 antigen. This may indicate that cardiac lymphatics may derive from the earliest cardiac veins. On the other hand, Prox-1^+^/Lyve-1^−^ strands of cells, clusters, and finally tubules were found to invade the great arteries from the mediastinum of mouse fetuses at 12-13 dpc (Figure 2) and Prox-1^+^/QH1^+^ strands of cells of quail fetuses at HH 26–36 stages, respectively [24, 25]. Contrary to what has been described in quail hearts the Prox-1-positive cells spread simultaneously on the arterial pole and on the atrial surface (the ventral and the dorsal aspect) of the developing mouse heart. These cells formed clusters and strands and subsequently, that is, at later stages of development, tubules/vessels that were CD31 and Lyve-1-positive (Figure 3) [25]. The mouse cardiac lymphangiogenesis proceeded on both surfaces of the heart: dorsal and ventral towards the apex in later stages of development. Recent studies indicate the coxsackie- and adenovirus receptors (CAR) as important factors in the formation of cell-cell adhesion and junctions between LEC in the mouse heart [51].

**Figure 4:**
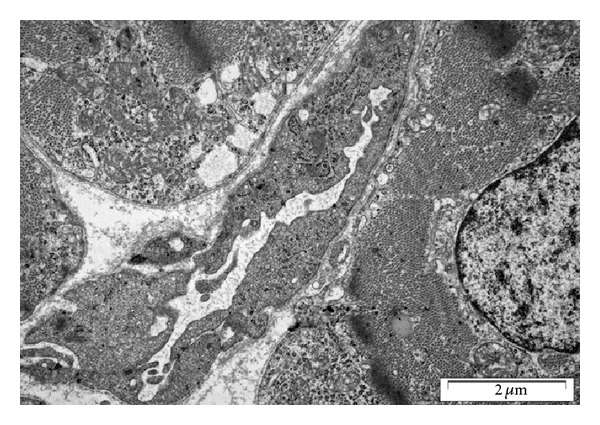
Ultrastructure of a developing subepicardial lymphatic vessel in a 4-day postnatal mouse heart. A tortuous appearance of LEC and anchoring filaments are visible.

It has been demonstrated by our group that cells bearing macrophage phenotypes were incorporated to lymphatic vascular walls during prenatal and early postnatal development of cardiac mouse lymphatics [25]. The exact phenotypes and the role of these cells in cardiac lymphatic vessel formation have not been elucidated. Postnatal development of cardiac lymphatic vessels in mice proceeded by sprouting from the subepicardial vessels towards the myocardium with a base-to-apex gradient. In fully developed hearts of other species the lymphatics are positioned along the branches of coronary arteries (adjacent to coronary arteries), similar to human hearts [22]. The ultrastructure of young, developing LEC is similar to that of the coronary vessel endothelium (Figure 4). The ultrastructure of fully developed lymphatic endothelium is characterized by the presence of many cisternae of Golgi complexes, pinocytotic vesicles, vacuoles, cytoplasmic microfilaments and anchoring filaments, and the lack of basement membrane [52, 53].

## 6. Concluding Remarks

An immense interest in biology and development of cardiac lymphatic vessels is nowadays justified because they are involved in normal functioning of the cardiovascular system and play a role in various pathologies. Understanding interspecies differences in normal structure and development of lymphatics is important for comparison of those structures in transgenic animals. Animal models with modification of selected genes help pursue a developmental process of lymphatic vessel formation and its regulatory mechanisms. Our recent work on mouse cardiac lymphatics using multiple markers of the lymphatic endothelium points to interspecies differences in capillary plexus location and in the course of major draining vessels. The lymphatics of a normal adult mouse heart, contrary to those in other mammalian species, are restricted to the outer zone of the myocardial wall (as has been previously suggested by Böger and Hort with the use of old techniques) [32]. Two major draining vessels have been identified: one, corresponding to the left collector in other species, runs along the left conal vein towards the left side of the pulmonary trunk and further to the mediastinum. The second vessel collects lymph from the left ventricle and runs on the surface of the coronary sinus towards the upper mediastinum. Recently published review articles [1, 4, 54] and the monographic edition by Karunamuni [10] summarize normal anatomy and structure of lymphatics, point to the newest techniques for studying cardiac lymphatics, the importance of the lymphatic system in tumor progression and metastasis, and describe the mechanisms of lymphangiogenesis in prenatal development and in various cardiovascular disorders.
